# Basal ganglia and cortical networks for sequential ordering and rhythm of complex movements

**DOI:** 10.3389/fnhum.2015.00421

**Published:** 2015-07-27

**Authors:** Jeffery G. Bednark, Megan E. J. Campbell, Ross Cunnington

**Affiliations:** ^1^Queensland Brain Institute, The University of QueenslandSt. Lucia, QLD, Australia; ^2^School of Psychology, The University of QueenslandSt. Lucia, QLD, Australia

**Keywords:** high-resolution fMRI, motor control, motor rhythm, motor order, multi-voxel pattern analysis, basal ganglia

## Abstract

Voluntary actions require the concurrent engagement and coordinated control of complex temporal (e.g., rhythm) and ordinal motor processes. Using high-resolution functional magnetic resonance imaging (fMRI) and multi-voxel pattern analysis (MVPA), we sought to determine the degree to which these complex motor processes are dissociable in basal ganglia and cortical networks. We employed three different finger-tapping tasks that differed in the demand on the sequential temporal rhythm or sequential ordering of submovements. Our results demonstrate that sequential rhythm and sequential order tasks were partially dissociable based on activation differences. The sequential rhythm task activated a widespread network centered around the supplementary motor area (SMA) and basal-ganglia regions including the dorsomedial putamen and caudate nucleus, while the sequential order task preferentially activated a fronto-parietal network. There was also extensive overlap between sequential rhythm and sequential order tasks, with both tasks commonly activating bilateral premotor, supplementary motor, and superior/inferior parietal cortical regions, as well as regions of the caudate/putamen of the basal ganglia and the ventro-lateral thalamus. Importantly, within the cortical regions that were active for both complex movements, MVPA could accurately classify different patterns of activation for the sequential rhythm and sequential order tasks. In the basal ganglia, however, overlapping activation for the sequential rhythm and sequential order tasks, which was found in classic motor circuits of the putamen and ventro-lateral thalamus, could not be accurately differentiated by MVPA. Overall, our results highlight the convergent architecture of the motor system, where complex motor information that is spatially distributed in the cortex converges into a more compact representation in the basal ganglia.

## Introduction

In order to perform skilled actions, such as playing a musical instrument, individual movements must be precise in their execution and timing. During skilled action performance, the brain must determine both the sequential order and individual timing of each movement, requiring neural processes for temporal and ordinal aspects of complex movement to be highly integrated. Given the close coupling of temporal (including rhythm) and ordinal processes in the production of fine motor skills, the nature of how temporal and ordinal movement information is represented in the brain has been long debated. It may be that temporal or rhythmic features of skilled movements are represented independently, or in an integrated fashion with the ordinal features (Conditt and Mussa-Ivaldi, 1999; Shin and Ivry, 2002; Ullén, 2007; O’Reilly et al., 2008; Ali et al., 2013; Kornysheva et al., 2013; Kornysheva and Diedrichsen, 2014).

Behaviorally, there is evidence for temporal and ordinal information being stored both in an integrated representation (Shin and Ivry, 2002; O’Reilly et al., 2008) and an independent representation (Ullén and Bengtsson, 2003). Supporting the notion of an integrated representation, O’Reilly et al. (2008) has shown that the cost of changing the order was as high as changing both order and timing, thereby indicating that the temporal features of a trained sequence cannot be transferred to a new sequence with a new order. Conversely, Ullén and Bengtsson (2003) have shown that learning a temporal structure can help facilitate the acquisition of a new order of sequential movements. Recently, a theory has been proposed that merges these different accounts (Kornysheva et al., 2013). According to Kornysheva et al. (2013), independently represented temporal and ordinal information is integrated in a multiplicative fashion when new action sequences are acquired. As a result, performance advantages associated with the initial temporal structure can only occur once the new ordinal representation has been formed.

Evidence from previous functional imaging studies indicate that partially independent brain networks control temporal and ordinal aspects of complex movement (Catalan et al., 1998; Schubotz and von Cramon, 2001; Tanji, 2001; Dhamala et al., 2003; Bengtsson et al., 2004; Garraux et al., 2005; Ullén, 2007; Bortoletto and Cunnington, 2010). In particular, different cortical regions are activated when sequences are created based on movement order compared to when the movement sequence is based on a rhythmic structure (Schubotz and von Cramon, 2001; Bengtsson et al., 2004; Ullén, 2007). For example, a fronto-parietal network, consisting of lateral prefrontal and inferior parietal areas, the basal ganglia and the cerebellum, has been shown to be more active when ordinal aspects are emphasized (Bengtsson et al., 2004). More temporal aspects of movement have been shown to engage the pre-supplementary motor area (SMA), the right inferior frontal gyrus and precentral sulcus, and the bilateral superior temporal gyri (Bengtsson et al., 2004).

This emphasis on identifying the distinct brain regions for temporal and ordinal movement features, however, misses the possibility that temporal and ordinal features may be represented in an integrative fashion in other brain regions. While most neuroimaging studies were designed to test for differences between complex movement processes, they did not focus on identifying the brain regions that were commonly active. In contrast, Garraux et al. (2005) specifically focused on identifying the brain regions that were commonly activated by temporal and ordinal processes, and subsequently employed functional connectivity to probe the nature of this representation. Their results indicated that temporal and ordinal processes could only be differentiated by the functional interactions between commonly activated regions and associated regions, namely the basal ganglia for timing and the cerebellum for sequential ordering (Garraux et al., 2005). More recent studies, employing multi-voxel pattern analysis (MVPA) techniques, have shown a hierarchical arrangement for the separation and integration of temporal and ordinal features of movement (Wiestler and Diedrichsen, 2013; Kornysheva and Diedrichsen, 2014). In particular, it has been shown that the representations of temporal and spatial features of movement are distinguishable within high-order premotor areas, but integrated within the primary motor cortex (Kornysheva and Diedrichsen, 2014). Recently, electrophysiological recordings from monkeys have shown that there are neurons within the premotor cortex that respond to both the temporal interval and the ordinal structure of a sequence of rhythmic movements (Merchant et al., 2013). This would indicate that even in commonly active brain regions, rhythmic and ordinal information may be represented independently.

In addition to the cortical motor regions, the basal ganglia also directly contribute to the acquisition and performance of action sequences (Lehéricy et al., 2005; Wymbs et al., 2012). While previous neuroimaging studies have delineated independent and integrative networks for complex movements at the cortical level, less is known about the representation of complex movement processes within the basal ganglia. Difficulties with both ordering movements (Robertson and Flowers, 1990; Fama and Sullivan, 2002; Smiley-Oyen et al., 2006) and the temporal rhythm of movement (Nakamura et al., 1978; O’Boyle et al., 1996) have been observed in individuals with Parkinson’s disease, in which the circuitry of the basal ganglia is greatly impaired (Obeso et al., 2008). The direct contribution of the basal ganglia to ordinal and rhythmic motor process has been demonstrated in several functional imaging studies (Dhamala et al., 2003; Bengtsson et al., 2004; Boecker et al., 2008; Orban et al., 2010). However, constraints of standard resolution images (which are insufficient when specifically examining basal ganglia activity) and conflicting accounts of studies linking basal ganglia activation to sequencing (Bengtsson et al., 2004) and timing (Garraux et al., 2005) has made it difficult to determine how rhythmic and ordinal processes are represented in the basal ganglia. However, there is emerging evidence indicating that the basal ganglia has a predominate role in temporal processes (Garraux et al., 2005; Wymbs et al., 2012; Kung et al., 2013). To better resolve motor process within the basal ganglia, high-resolution 3T imaging has been employed to identify the representation of basic movement parameters such as movement frequency, movement selection, and sequence complexity within segregated basal ganglia-cortical circuits (Lehéricy et al., 2006; Mattfeld et al., 2011). Here, we use high-resolution 3T functional Magnetic Resonance Imaging (fMRI) imaging to better examine complex movements relating to the sequential rhythm and sequential order of movement within basal ganglia-cortical circuitry.

The present study employed instructed, discrete finger movement sequences in order to probe the representation of rhythmic and ordinal features in commonly activated brain regions within the basal ganglia and thalamus. Unlike continuous reaching movements, in which the temporal profile appears tightly bound with the learnt spatio-temporal trajectory (e.g., Conditt and Mussa-Ivaldi, 1999), discrete action sequences appear to have independent temporal and ordinal representations, at least within the cortex (e.g., Bengtsson et al., 2004; Ullén, 2007; Kornysheva and Diedrichsen, 2014). Thus, using discrete finger movements would allow for maximal dissociation between sequential rhythm and order.

In addition to examining activation within the basal ganglia-cortical circuitry, we also applied MVPA methods to try to dissociate and classify activity within the basal ganglia and within cortical brain regions commonly activated by both sequential rhythm and sequential ordering. Compared with univariate approaches which measure changes in the *mean* signal intensity across spatially smoothed voxels (Friston et al., 1994), MVPA is sensitive to signal *variability* or spatial “pattern” across a region of voxels (Jimura and Poldrack, 2012; Davis et al., 2014). As a result, MVPA can detect differences in coding of task-relevant information within regions based on differing patterns across voxels rather than overall changes in mean activation level (Mur et al., 2009). Thus, MVPA allows for investigation of whether activity in regions that show common activation across conditions may reflect functionally separable underlying processes (Peelen et al., 2006; Peelen and Downing, 2007).

The aim of the present study was to use high-resolution fMRI and multivariate analysis techniques to investigate whether the representation of complex movement processes, sequential rhythm and sequential order, can be disentangled within the basal ganglia-cortical networks and brain regions that are commonly activated for both types of complex movements. To examine complex movements with increased demand on sequential rhythm and sequential order processes we employed three different finger-tapping tasks: (1) a simple, single finger-tapping task; (2) a complex, single finger-tapping task with a varying temporal rhythm between movements; and (3) a complex finger-tapping task with all four fingers tapping in sequential order but no variation in temporal rhythm. Compared with the simple movements, each of the complex movements involved increasing demand on the temporal rhythm or sequential ordering of submovements. Based on previous work, we hypothesize that the supplementary motor, inferior frontal gyrus, and basal ganglia will be more active for rhythmic movements, and high-order planning areas such as the premotor cortex and parietal brain regions will be more active during the ordinal sequencing of movements. Within brain regions that are commonly activated by rhythmic and ordinal tasks, we will use multivariate classification techniques to probe the representation of these complex movement features.

## Materials and Methods

### Participants

The participants were 20 healthy young volunteers (11 females and 9 males; mean age ± SD: 23.8 ± 3.2 years) who gave their written informed consent. All participants were right-handed according to the Edinburgh Handedness Inventory (Oldfield, 1971). The Medical Research Ethics Committee of the University of Queensland approved all procedures.

### Tasks

**Figure 1:**
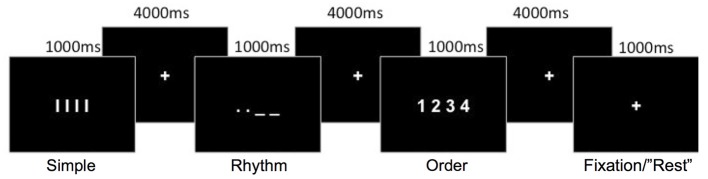
**Schematic representation of the movement conditions.** Each movement condition was preceded by a visual cue indicating the movement sequence to perform. A rest condition involved the fixation-cross presented for an extra 1000 ms.

In three separate movement conditions (Figure 1), participants were instructed to perform short, self-paced finger movements using their right hand. Finger movements were performed by making finger presses on an fMRI-compatible four-button response box. The three conditions differed in temporal rhythm and ordinal sequence complexity, as follows:

1. Simple: Participants were instructed to use their right index finger to perform four simple repetitive finger presses, at a comfortable rapid speed, without any variation in the temporal rhythm of the movements. All four finger-presses were performed on the first button. This Simple finger-press condition served as a baseline comparison for the Rhythm and Order conditions.
2. Rhythm: Similarly to the Simple movement condition, participants were instructed to perform four repetitive finger movements with their right index finger. To introduce a rhythmic timing pattern to the movements, participants were visually cued with a single image (see Figure 1) to vary the timing of presses between long and short, in Morse code fashion. This visual cue presented the timing for the four sub-movements that the participants were to perform prior to the onset of the sequence; they were not cued separately prior to each single finger movement. Therefore, in contrast to the Simple condition, the finger-presses had to be coordinated with a specific temporal rhythm.
3. Order: Participants were instructed to make four sequential finger presses on all four buttons using all four fingers (1-index, 2-middle, 3-ring and 4-little finger) in a designated order. Movement sequences always began with the index finger pressing the first button, and the required sequential order was visually presented (see Figure 1) to the participants prior to movement initiation. Similar to the Simple condition, participants were instructed to make finger presses with repetitive timing. Thus, only the complexity of the ordinal movement sequence was increased in this condition, but not the demand on the temporal rhythm of finger-presses.

As shown in Figure 1, the required movement sequence for each of the three movement conditions was cued visually for 1000 ms, with a fixation-cross displayed for 4000 ms between each sequence cue. Movements were therefore cued at a rate of one every 5 s. A rest condition, in which participants did not perform any movement, was also included. This consisted of the fixation cross remaining displayed (1000 ms) instead of a movement cue and appeared to participants as a random long inter-trial interval. A single run consisted of 72 trials (18 trials per condition plus 18 rest trials) presented in one of two predetermined random sequences so that the order of the conditions was pseudorandomized. Each run lasted 6 min 8 s in duration. Four runs were conducted for each participant, with the order of the two random sequences counter-balanced between participants. One training run (72 trials) was conducted outside the scanner to familarize the participants with the task. During this practice run participants were instructed to emphasize accuracy over speed. The training was not intended for participants to learn the specific sequences, as the sequences during the fMRI task were cued on every trial, but served to familiarize participants with the task and for us to ensure that they properly understood the instructions.

### Behavioral Analysis

For each movement condition, behavioral performance was measured using three performance indices: mean reaction time, measured as the time between visual cue presentation and the first button press; mean movement duration, measured as the time between the first and the last button press; and mean percentage of correct responses. A trial was considered correct if all four finger-presses were performed with the correct temporal rhythm or sequential order. Specifically, for the Rhythm condition, accuracy was dependent on the timing of each movement within the sequence relative to the mean for the 4 button-presses, with short intervals required to be shorter than the mean and long intervals required to be longer than the mean.

Separate repeated-measures ANOVAs were conducted for each performance measure with the three levels of *Condition* (Simple, Rhythm, Order). The Greenhouse-Geisser correction was applied where appropriate, and *post hoc* comparisons were Bonferroni corrected for multiple comparisons. All behavioral data was tested for normality using the Shapiro-Wilk test. Non-normal data were transformed appropriately.

### fMRI Acquisition

fMRI data were acquired on a whole-body 3-Tesla Siemens Trio MRI scanner (Siemens Medical System, Germany) equipped with a 32-channel head coil. To minimize head movement, foam padding was placed securely and comfortably around the participant’s head prior to scanning. Functional images were acquired using a sequence designed to provide high-resolution images of the basal ganglia and thalamus, covering the top of the vertex to the top of the cerebellum (below the level of the basal ganglia and thalamus). To this aim, images were acquired using a Gradient echo echo-planar imaging (GE-EPI) sequence with the following parameters: 30 axial slices; echo time (TE) 31 ms; repetition time (TR) 2150 ms; parallel imaging (iPat) factor 2; flip angle (FA) 80°; pixel bandwidth 1682; Field of View (FOV) 192 × 192 mm and 128 × 128 voxel matrix; resolution 1.5 × 1.5 × 2 mm^3^ with 1 mm slice gap. Online geometric distortion correction (DiCo) was applied on the basis of the point-spread function data acquired before the EPI scans using a specialized sequence (Zaitsev et al., 2004). A total of 171 EPI images were acquired for each of the four runs. The first three EPI images of each run were removed to obtain stable magnetization. A three-dimensional high-resolution T1-weighted structural image covering the entire brain was also acquired for anatomical reference (TE = 2.32 ms, TR = 1900 ms, FA = 9°, 256 × 256 cubic matrix, voxel size = 0.9 × 0.09 × 0.9 mm^3^).

### fMRI Preprocessing

All fMRI data were preprocessed and analyzed using Statistical Parametric Mapping (SPM8; Wellcome Department of Imaging Neuroscience, Institute of Neurology, London, UK),^1^ implemented in Matlab (Mathworks Inc., Natick, MA, USA). Functional images were first realigned temporally using slice-timing correction procedures (Sladky et al., 2011). To correct for head movement, the images were then spatially realigned with reference to the inner most slice using a six-parameter rigid body spatial transformation. The structural T1-image was then co-registered to the mean functional image obtained during realignment. The co-registered T1-image was used to derive the transformation parameter to register the functional images to standard Montreal Neurological Institute (MNI) stereotaxic space using the segmentation function in SPM8. To maintain the high-resolution of the acquired images, the functional images were re-sliced to a resolution of 1 × 1 × 1 mm^3^ and smoothed using a 3 mm full-width half maximum (FWHM) isotropic Gaussian kernel. The T1-image was also re-sliced to a resolution of 0.5 × 0.5 × 0.5 mm^3^ for display purposes. An average group anatomical image was created using the T1-images from each participant registered to MNI space.

### Univariate fMRI Activation Analysis

The fMRI data were analyzed using a general linear model (GLM) and event-related design. Neural activity (blood oxygen level dependent; BOLD signal) for the three movement conditions were separately modeled with a hemodynamic response function with a 1 s duration, using a 60 s high-pass temporal filter. Onset times for modeling the different movement conditions were linked to the onset of the visual cue indicating to the participant the sequence of movements to perform. Trials for the Rest condition were not explicitly modeled, as they appeared to participants simply as a longer inter-trial interval, and contributed to an implicit baseline in the GLM analysis. Estimates of activation from the single-subject models for each movement condition contrasted with the implicit baseline were used to construct the group-level analysis.

At the group-level, contrast estimates for each of the three conditions for each participant were entered into a single factorial model within SPM8. Activation maps for each of the movement conditions were first contrasted with the implicit baseline (Simple, Rhythm, and Order). For these movement conditions we report results using a voxel-level corrected threshold *P*_FWE_ < 0.05 and 20 voxel cluster extent. We used this voxel-level threshold to limit the large amount of activity when each of the movement conditions was compared to the implicit (resting) baseline. To identify brain activity common to all movement conditions, a conjunction null analysis (Nichols et al., 2005) was conducted across these three contrasts.

For brain activity associated with complex movements (Rhythm and Order conditions) relative to simple movements (Simple condition), a further conjunction null analysis was conducted across contrasts of Rhythm-Simple and Order-Simple. To specifically examine the difference between sequential rhythm and sequential order, activation maps for the Rhythm and Order conditions were directly contrasted (Rhythm-Order and Order-Rhythm). A cluster-level threshold of *P*_FWE_ < 0.05 (clusters defined by voxel-level threshold *p* < 0.001) was used for these analyses.

In order to examine activation within the basal ganglia and thalamus in more detail, each of the above contrasts were also conducted with a small-volume correction using a basal ganglia and thalamus anatomical mask defined using the Automated anatomical labelling (AAL) brain atlas (Tzourio-Mazoyer et al., 2002) and conducted using the Wake Forest University (WFU) Pickatlas toolbox.^2^ A cluster-level threshold of *P*_FWE_ < 0.05 (clusters defined by voxel-level threshold *p* < 0.001) was also used for these small-volume corrected analyses.

### Multi-Voxel Pattern Analysis (MVPA)

We performed MVPA to determine if brain regions commonly activated by sequential rhythm and sequential order encoded functionally distinct information (Haxby et al., 2001; Peelen et al., 2006; Peelen and Downing, 2007). Before conducting pattern classification analysis, a GLM was estimated using slice-timing and motion-corrected functional data. The data was left non-normalized and unsmoothed in order to maximize the amount of information contained in the spatial activation patterns (Cox and Savoy, 2003). Similar to the GLM conducted for the univariate analysis, this GLM yielded parameter estimates for each of the conditions (vs. the implicit baseline) for each participant and each run.

Searchlight decoding analysis, using the Decoding Toolbox beta version (Görgen et al., 2012), was conducted using the parameter estimates from the Rhythm and Order conditions. The “searchlight” decoding approach was used to probe the information content in local spatial clusters around every voxel (Bode et al., 2012). Specifically, each voxel in the brain served as a central voxel around which a searchlight cluster was constructed with a 9 mm radius (Bode and Haynes, 2009). Parameter estimates from voxels within each “searchlight” cluster from each participant, condition (Rhythm or Order), and run were extracted and entered into a linear support vector (LSV) classifier, performed using the LIBSVM toolbox.^3^ A four-fold cross-validation design was used for training and testing the LSV classifier. Specifically, parameter estimates from three out of the four fMRI runs were selected to serve as the training data-set used to train the classifier to determine an optimal linear hyperplane for classifying Rhythm vs. Order conditions. The remaining fMRI run served as the test data set and the optimal linear hyperplane was subsequently used to classify trials as Rhythm or Order in this independent test run. This process was repeated in four steps until each fMRI run served once as the test set, with the classifier trained on all combinations of the other three runs (four-fold cross-validation). Three-dimensional accuracy maps were created from the mean probability of correct classification (accuracy minus 50% chance level) that was assigned to each central voxel of a searchlight cluster by the support vector classifier (SVC; Soon et al., 2008; Bode and Haynes, 2009; Bode et al., 2012).

The decoding accuracy maps were normalized to MNI space and smoothed with a 3 mm FWHM Gaussin kernel, using the same parameters as calculated during preprocessing for the univariate analysis. For group analysis, these classification accuracy maps were analyzed by single-sample *t*-test in SPM8 (i.e., testing the difference from chance-level 50% classification accuracy). In order to specifically examine differences in brain regions that were commonly activated by both sequential rhythm and sequential order, second-level analysis of MVPA accuracy map were conducted only on voxels within regions that were significantly active in the univariate conjunction of Rhythm-Simple and Order-Simple conditions. We did this by applying a functional mask based on activations from the conjunction null analysis. To examine encoding of sequential rhythm and sequential order in the basal ganglia in more detail (i.e., with increased statistical sensitivity), we also performed a small-volume corrected analysis including only voxels that were significant in the univariate conjunction of Rhythm-Simple and Order-Simple conducted within the basal ganglia-thalamus anatomical mask previously used for univariate analysis. All analyses of group level decoding accuracy maps used a cluster-level threshold of *P*_FWE_ < 0.05 (clusters defined by voxel-level threshold *p* < 0.001).

We also used the Decoding Toolbox to conduct region of interest (ROI) MVPA considering the spatial pattern of activity across all voxels within the basal ganglia-thalamus anatomical mask, rather than by a voxel-wise fashion performed by searchlight MVPA. While this form of analysis limits our ability to localize areas that differentially encode movement conditions, it allows us to test with greater sensitivity if any patterns of activation, across the entire basal ganglia and thalamus, can accurately differentiate between Rhythm and Order conditions. Thus, whole-region ROI MVPA allows us to test whether any classification between the two complex movement conditions was possible within these subcortical pathways irrespective of location.

For this ROI decoding, an explicit mask of the basal ganglia and thalamus was applied to the functional scans prior to the first-level GLM analysis using normalized, but unsmoothed data. Parameter estimates within the explicit basal ganglia-thalamus ROI for each participant, condition (Rhythm or Order), and run were entered into the SVC to perform leave-one-run-out cross validation decoding analysis, similar to that described above for the searchlight analysis. For each participant, a single probability of correct classification (accuracy minus 50% chance level) for the entire basal ganglia-thalamus ROI was obtained. To determine if the probability of correct classification was significant within the basal ganglia-thalamus ROI, we conducted a one-sample *t*-test on classification scores.

For both univariate activation maps and multivariate accuracy maps, the anatomical locations of brain activity were initially identified using the AAL brain atlas toolbox and MRIcron.^4^ More specific anatomical labels including the Brodmann areas (BAs) and recently defined anatomical labels of regions additional to the defined BAs were identified using the SPM Anatomy toolbox (Eickhoff et al., 2005). For thalamic subregions, we used the seven connectivity zones defined by Behrens et al. (2003).

## Results

### Behavioral Results

**Figure 2:**
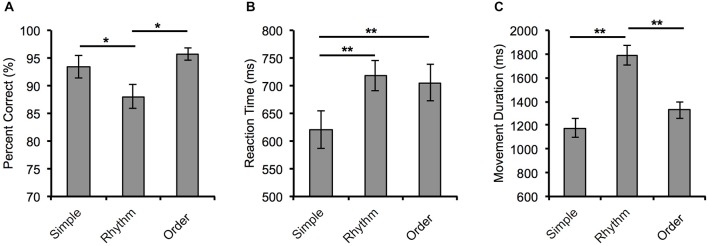
**Behavioral measures of performance for the three movement conditions. (A)** Mean percentage correct, **(B)** reaction time, and **(C)** movement duration for each movement condition. Error bars = ±SEM. *p < 0.05 and **p < 0.001.

The behavioral data are shown in Figure 2. Our analysis of the behavioral data revealed a significant main effect of *Condition* for each of the three performance indices: Percentage correct *F*_(2,38)_ = 6.64, *p* = 0.04, *Partial η*^2^ = 0.259); reaction time *F*_(2,38)_ = 26.81, *p* < 0.001, *Partial η*^2^ = 0.585); and movement duration *F*_(2,38)_ = 32.15, *p* < 0.001, *Partial η*^2^ = 0.629).

Specifically, participants’ accuracy in the Rhythm condition (88.06 ± 2.24%) was significantly reduced compared to performance in the Order (95.38 ± 1.09%, *p* = 0.005) and Simple conditions (93.47 ± 2.08%, *p* = 0.04). Participants were significantly faster at reaction to the visual cue in the Simple condition (620 ± 34 ms) than in the Rhythm condition (718 ± 28 ms, *p* < 0.001) and the Order condition (705 ± 33 ms, *p* < 0.001). Movement duration in the Rhythm condition was significantly longer (1792 ± 85 ms) than in both the Simple condition (1176 ± 79 ms, *p* < 0.001) and the Order condition (1329 ± 71 ms, *p* < 0.001).

### fMRI Whole-Brain Activation Results

Activations from each of the three movement conditions were tested relative to baseline. There was significant activation in motor regions of the left hemisphere that was common across the three movement conditions. In the left cortex, activity was found in the primary motor cortex (BA 6, 4), primary somatosensory cortex (BA 3, 2), inferior parietal lobule (IPC), supramarginal gyrus (OP1), and insula cortex Subcortically, there was activation in central portions of the left putamen and the left thalamus within the dorsal medial nucleus. There was also bilateral activation in the SMA (BA 6) and in task-relevant regions in the visual cortex for viewing and recognising visual cues.

**Figure 3:**
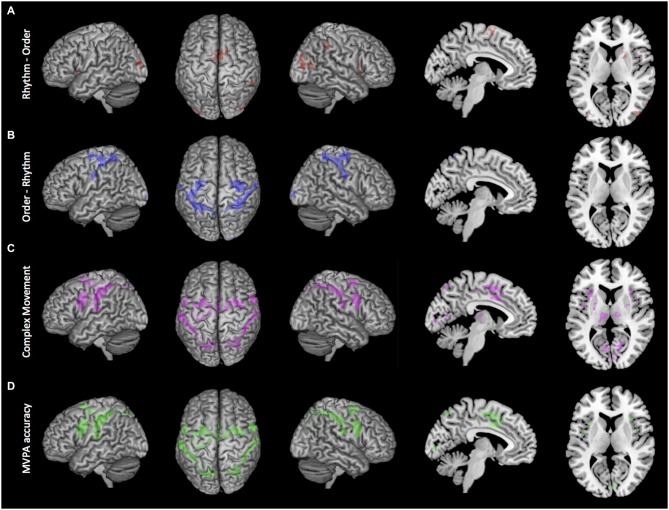
**Brain active brain regions and classification accuracy maps. (A)** Order-Rhythm contrast demonstrated significant activity in the lateral premotor areas, primary somatosensory cortex, and superior parietal lobule. **(B)** Rhythm-Order contrast demonstrated significant activity in the supplementary motor area and right putamen and caudate. **(C)** Conjunction null analysis of complex movement (Rhythm-Simple and Order-Simple) demonstrated significant activity in medial premotor cortex, lateral premotor areas, and inferior and superior parietal lobes. **(D)** Whole-brain searchlight decoding of Rhythm vs. Order demonstrated that brain regions with significant classification of sequential rhythm and sequential order closely match the regions showing common activation for Complex movement. Clusters reflect voxels that exceed cluster-level threshold *P*_FWE_ < 0.05 and are superimposed onto a MNI atlas brain.

Conjunction analysis was used to examine the shared activity of the two complex movement conditions (Rhythm and Order conditions) against the Simple movement condition. Several brain regions revealed significant activation that was common for both of the complex movement conditions (Figure 3). There was an extensive cortical network that included bilateral activation in the superior frontal gyrus (BA 8), medial premotor cortex including the supplementary motor area (BA 6) and the cingulate motor area (BA 24), the lateral premotor area (BA 6), insula (BA 13, 14), inferior frontal gyrus (BA 44), primary motor cortex (BA 4), primary somatosensory cortex (BA 2), supramarginal gyrus (OP1, OP4), and inferior (IPC, hIP2, hIP3) and superior parietal lobule (7A, 7P, 7PC). In subcortical regions we found bilateral activation within central portions of the putamen and in the thalamus mostly in ventral lateral and dorsal medial nuclei, but also extending through ventral posterior lateral and ventral posterior medial nuclei, as well as activity in the cerebellum. Also, compared to the Simple movement condition, there was greater activation in the visual cortex that was likely associated with more complex visual cues present at the start of the Rhythm and Order conditions.

To identify brain regions that had greater activation during sequential rhythm, we directly compared the Rhythm condition with the Order condition (Figure 3). Sequential rhythm activated a predominately prefrontal-supplementary motor area-basal ganglia network. Specifically, we found greater bilateral activation of the inferior frontal gyrus (BA 44) and the supplementary motor area (BA 6) for the Rhythm condition. In the right hemisphere there was greater activation in the insula (BA 13, 14), supramarginal gyrus (PFm), inferior parietal lobule (PFm) and middle temporal gyrus (PGp). Subcortically, we found more activation in the right putamen and the head of the right caudate. There was also more activation in the middle occipital lobe.

The Order condition was then contrasted against the Rhythm condition to find brain regions that had greater activation for sequential order. As shown in Figure 3, sequential order activated predominantly a bilateral premotor-parietal cortical network. This included bilateral activation of the lateral premotor area (BA 6), primary somatosensory cortex (BA 3, 2, 1), and inferior (PFt) and superior parietal lobule (7A, 7P, 7PC, 5L). In the left hemisphere there was more activation in the primary motor cortex (BA 4), while in the right hemisphere there was greater activation in the right rolandic operculum (OP1, OP4). There was also greater activation in the visual cortex (BA 17, 18).

### Basal Ganglia-Thalamus Results

**Figure 4:**
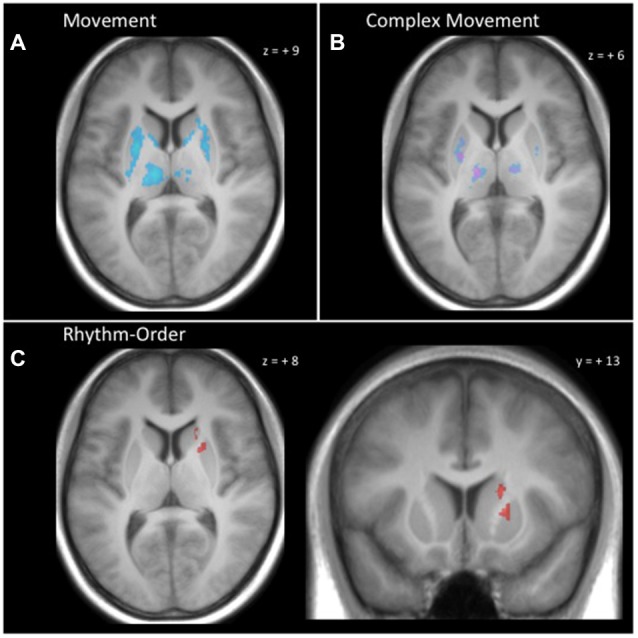
**Basal ganglia and thalamus activity. (A)** Conjunction null analysis across all movement conditions (Simple, Rhythm, Order) demonstrated common activation bilaterally in the putamen, caudate and thalamus. **(B)** Conjunction null analysis for complex movements (Rhythm-Simple and Order-Simple) demonstrated greater common activation in the putamen and thalamus bilaterally. **(C)** Rhythm-Order contrast demonstrated greater activation for Rhythm in central dorsomedial portions of the right putamen and central dorsolateral portions of the right caudate. Clusters reflect voxels that exceed cluster-level threshold *P*_FWE_ < 0.05 and are superimposed onto a single participant’s T1 image.

As shown in Figure 4, the performance of actions in the three movement tasks involved extensive activation in the basal ganglia and thalamus compared to baseline. This activity was mostly within the known motor circuit of the basal ganglia. Specifically, there was extensive bilateral activity in the putamen, caudate and thalamus that was common to all three movement tasks. This extensive activity was reduced when testing the conjunction of complex movement conditions (Rhythm and Order) compared with the Simple condition. For the complex movements, common activation was found bilaterally in central portions of the putamen and thalamus (Figure 4).

Making use of the high-resolution images of the basal ganglia and thalamus, we were able to identify distinct portions of the caudate and putamen that were more active during the Rhythm condition compared to the Order condition. This distinct activity can be seen in Figure 4. We found that central dorsomedial portions of the right putamen (MNI: 20, 14, 2) and central dorsolateral portions of the right caudate (MNI: 18, 23, 5) were more active for sequential rhythm. Interestingly, when the Order condition was contrasted against the Rhythm condition, we did not find any regions of the basal ganglia or thalamus that were more active.

### Movement Duration Correlation Analysis

Behavioral data showed that movement durations were significantly longer in the Rhythm condition compared with other conditions. To test whether this could account for activation differences between Rhythm and Order conditions, we examined the correlation between movement durations and activation differences for the Rhythm condition across participants. We first extracted each participant’s beta estimates for peak activation voxels in brain regions that were significantly more active for the Rhythm compared to the Order conditions. Correlation analysis was then conducted to determine if there was any significant correlation between these beta estimates and the difference in movement duration between each condition across participants. This analysis revealed no significant correlations in any of the active brain regions, indicating that the activation differences between Rhythm and Order conditions could not be accounted for simply by the difference in movement duration between the two conditions.

### MVPA Results

As highlighted above, conjunction null analysis revealed an extensive cortical and subcortical network that was commonly activated by sequential rhythm and sequential order (Figure 3). However, MVPA revealed that the patterns of activation in most of these commonly activated cortical regions reflected functionally dissociable processes mediating sequential rhythm and sequential order (Figure 3, lower panel). Specifically, we found that patterns of activation in bilateral portions of the supplementary motor area (BA 6), the cingulate motor area (BA 24), the lateral premotor area (BA 6), the insula (BA 13, 14), the inferior frontal gyrus (BA 44), primary motor cortex (BA 4), the primary somatosensory cortex (BA 2), supramarginal gyrus (OP1, OP4), and the inferior (IPC, hIP2, hIP3) and superior parietal lobule (7A, 7P, 7PC) could differentiate functional activity for sequential rhythm and sequential order. Thus at the cortical level, sequential rhythm and sequencing processes could be decoded in all the regions showing common activation across both conditions.

Interestingly, patterns of activation for sequential rhythm and sequential order could not be decoded in commonly activated portions of the basal ganglia-thalamus. Searchlight decoding analysis in commonly active voxels conducted at the whole-brain level (Figure 3, lower panel) and in commonly active voxels within the basal ganglia and thalamus (small-volume corrected) did not find any portions of the basal ganglia or thalamus that could accurately differentiate sequential rhythm and sequential order. Even whole-region ROI decoding analysis considering all voxels across the entire basal ganglia and thalamus did not find activation patterns that differentially encoded sequential rhythm and sequential order (mean decoding accuracy: 53.13 ± 11.38%; *t*_(19)_ = 1.23, *p* = 0.234).

## Discussion

In the present study, we used high-resolution fMRI and multivariate analysis techniques to examine the representation of complex movement processes within basal ganglia-cortical networks and commonly activated brain regions. At the level of the cortex, we found that complex movements with different demands on the rhythmic timing and sequential order of submovements could be distinguished both by the different brain networks they recruited and by the spatial patterns of activity within commonly activated brain regions. Within the basal ganglia, complex movements commonly activated distinct portions of the basal ganglia to a greater extent than simple movements, with movements involving a complex sequential rhythm demonstrating greater activity in the right putamen and caudate than movement with complex ordinal sequences. MVPA, however, could not dissociate activity for rhythm and order conditions within those regions of the basal ganglia and thalamus commonly activated for both complex movements.

We found that discrete finger movements with a complex rhythm activated the SMA and the right basal ganglia to a greater extent than movements with a complex ordinal sequence. This finding of greater SMA and basal ganglia activity for rhythmic movement features is inline with the view that the SMA and basal ganglia are key nodes for rhythmic and temporal processing (Ferrandez et al., 2003; Buhusi and Meck, 2005; Macar et al., 2006; Karabanov et al., 2009; Wiener et al., 2010; de Manzano and Ullén, 2012; Kung et al., 2013; Yin, 2014). The importance of these brain regions has been further highlighted by studies with Parkinson’s Disease (PD) patients and patients with SMA and basal ganglia lesions, who show decrease ability to perform rhythmic movements (Halsband et al., 1993; Koch et al., 2008; Coslett et al., 2010). The SMA and basal ganglia are anatomically and functionally connected (Cavada and Goldman-Rakic, 1991; Nachev et al., 2008; Habas, 2010; Zhang et al., 2012). Both the SMA and basal ganglia appear to perform important clock functions in the brain, with the SMA thought to be a key node of the putative clock mechanism (Macar et al., 2006; Wiener et al., 2010) and the basal ganglia’s role in timing is thought to stem from the striatal beat frequency model that proposes a biologically plausible account of timing (Matell and Meck, 2004). Relating to the present study, in which visual information was used to instruct the rhythmic task, Mita et al. (2009) has shown that neurons in the monkey SMA contribute to inference of temporal constraints from visual instructions. Additionally, we found greater activation in the inferior frontal gyrus and right inferior parietal regions, which have also been associated with temporal processes (Bueti and Walsh, 2009; Craig, 2009).

When the discrete finger movements involved a complex ordinal pattern we found greater activation in the lateral premotor cortex and inferior and superior parietal regions. These brains regions appear to be most active when movements involve a high degree of sequential organization (Bengtsson et al., 2004; Ullén, 2007). Premotor brain regions are heavily connected with parietal regions, and both brain regions play a key role in sequential organization of movements in general (Jubault et al., 2007; Beudel and de Jong, 2009; Bortoletto and Cunnington, 2010). Interestingly, it appears that the performance of an unfamiliar discrete ordinal finger movement sequence involves different process from implicit sequence learning. For implicit sequence learning the basal ganglia has been shown to be a key brain region (Yin, 2010; Gheysen et al., 2011), but here we found the basal ganglia was more active for the rhythmic condition compared with the ordinal condition. Given that a temporal rhythm is thought to emerge during sequence learning (Sakai et al., 2004), it may be that the basal ganglia has a key role in creating the temporal signature of a learnt movement sequence. This process may relate to the known role of the basal ganglia in motor chunking (Graybiel, 1998; Yin, 2010; Wymbs et al., 2012). It has even been suggested that temporal information is integrated with the ordinal selection of movements through chunking (Gobel et al., 2011).

The activation of distinct brain regions for rhythmic and ordinal movement features is inline with the notion of independent representations for these two motor features. However, to fully understand how these two features are represented in the brain it is important to also assess their representation in brain regions that are commonly activated by the two features. To this aim we examined the level to which complex movements commonly activate overlapping brain regions and whether the representation of sequential rhythm and sequential order within overlapping brain regions can be differentiated using multivariate classification techniques. Both rhythmic and ordinal movement tasks commonly activated an extensive network extending from the superior frontal cortex and inferior frontal gyrus to the parietal cortex. Previous work investigating the regions commonly activated by timing-related and order-related motor processes found a similar cortical network (Garraux et al., 2005). Interestingly, Garraux et al. (2005) did not report any difference in cortical activity between the two sequence processes. In their study, both temporal and ordinal conditions involved a series of unknown sequential finger movements, using multiple fingers, with variable timing between movements. In the temporal condition, a sequence was performed twice as presented to the participant, but with participant-determined variation in timing between movements, while in the ordinal condition the presented sequence was performed once in reverse order and once in the same order. Thus, the differences between the two conditions were quite small. However, sequence timing and sequence order motor processes could be distinguished by their functional connectivity between shared brain regions and the putamen and cerebellum (Garraux et al., 2005). While their results indicate that the functional processes within commonly activated brain regions may be determined by their functional interaction with other brain regions, they could not assess whether the representation of motor timing and sequencing processes within overlapping brain regions were distinct.

Using MVPA, we probed the functional representation of complex motor processes within shared brain regions in order to determine if common activation was indicative more of an integrated representation or an independent representation. We found that search-light based MVPA could accurately classify patterns of activity associated with the sequential rhythm task from patterns of activity associated with the sequential order task within these commonly activated cortical regions. Specifically, MVPA showed significant discrimination between both types of complex movements in bilateral portions of the SMA, the cingulate motor area, the lateral premotor area, the insula, the inferior frontal gyrus, primary motor cortex, the primary somatosensory cortex, supramarginal gyrus, and the inferior and superior parietal lobule. These cortical regions either directly represent motor execution and somatosensory processes (primary motor and somatosensory areas), or are involved in more abstract representations of the temporal (SMA) and ordinal (e.g., lateral premotor, cingulate and parietal regions) structure of movement (Rushworth et al., 2001; Lewis and Miall, 2003; Bengtsson et al., 2004; Bortoletto and Cunnington, 2010). Based on the notion that MVPA can be used to interpret overlapping functional activations (Peelen and Downing, 2007), this would suggest that complex movement with differing rhythmic and ordinal demands may be mediated by functionally dissociable neural populations.

The independent albeit partly overlapping representation of rhythmic and ordinal features indicated by the present results would allow for the separate learning of temporal and ordinal features. This independent representation is thought to underlie behavioral findings indicating there are performance advantages for untrained movement sequences when a previous movement sequence has been acquired (Ullén and Bengtsson, 2003; Ali et al., 2013; Kornysheva et al., 2013; Kornysheva and Diedrichsen, 2014). Interestingly, Kornysheva and Diedrichsen (2014) have recently shown that when movement sequences with both temporal and ordinal features are learnt, the representation of the temporal and ordinal features is integrated in the primary motor cortex, but independent in higher motor regions. Our results merge with recent decoding studies to support a multiplicative account of temporal and ordinal representation (Kornysheva et al., 2013). It is important to note, however, that the independent representation of temporal and ordinal features may depend on the type of movement performed. In the present study, participants performed untrained and discrete sequential finger-tapping movements. Thus, an independent representation of rhythmic and ordinal features would seem likely. For movements that are more continuous, temporal and ordinal features are likely heavily integrated (Conditt and Mussa-Ivaldi, 1999) and their representation may be difficult to separate using multivariate classification techniques.

MVPA is known to be an “opportunistic” method, in that it can use any basis of difference between conditions on which to make a classification (e.g., Haynes and Rees, 2005; Johnson et al., 2009; Rissman et al., 2010; Watanabe et al., 2011). Thus, while we find that MVPA can accurately differentiate between sequential rhythm and sequential order within overlapping brain regions, classification may not be solely based on processes directly related to temporal rhythm or sequential order and so results must be interpreted with caution.

Motivated by the apparent difficulty of previous neuroimaging studies to differentiate the contributions of the basal ganglia to sequential rhythm and sequential order processes, we applied high-resolution fMRI to investigate the intricate functional architecture of the basal ganglia. In accordance with theoretical accounts of basal ganglia function, we found that all movement conditions commonly activated substantial portions of the putamen bilaterally and sections of the caudate, as well as ventrolateral portions of the thalamus. We found that complex temporal rhythm involved the preferential recruitment of portions of the right dorsomedial putamen and the right dorsolateral caudate nucleus. This right side bias of basal ganglia activity is consistent with previous findings that link basal ganglia activity with timing processes (Hinton and Meck, 2004; Garraux et al., 2005; Wiener et al., 2010). Importantly, the putamen together with the SMA form a large portion of the cortical-basal ganglia motor loop (Alexander et al., 1986). Thus, preferential activity within this loop may suggest that temporal processes engage a larger portion of the motor circuit.

We did not find any portion of the basal ganglia displaying significantly greater activation for the sequential order when contrasted with the rhythmic task. However, we found bilateral activation of the putamen when conjunctive null analysis was conducted with all three movement tasks and with the complex vs. simple movements (Rhythm-Simple with Order-Simple), though to a lesser extent for complex movements. Recently, bilateral activation of the basal ganglia has been suggested to be necessary for the strengthening of motor-motor associations (Wymbs et al., 2012). The common bilateral activity we found in the putamen would reflect the action chunking processes that the basal ganglia is thought to perform during the learning of movement sequences (Graybiel, 2008; Boyd et al., 2009; Tremblay et al., 2009; Yin, 2010).

In contrast to cortical regions, areas of overlapping activation in the basal ganglia and thalamus did not show differential patterns of activity for sequential rhythm and sequential order that could be classified using search-light MVPA. This finding was also supported when whole-region ROI based MVPA was applied. The difference in classification accuracy between cortical and subcortical regions has previously been noted (Jimura and Poldrack, 2012), and may be due to differences in how functional information is represented. It has been proposed that sensitivity differences in MVPA between cortical and subcortical brain region may be a factor of how information in structured, being more widely distributed in the cortex and more compact in subcortical nuclei (Jimura and Poldrack, 2012). Interestingly, our univariate analysis revealed areas of the caudate and putamen that were differentially active for rhythm compared with order conditions. While MVPA assesses the spatial structure of activation patterns to determine if a region contains information that can accurately decode different categories (Kriegeskorte et al., 2006), activation-based analysis uses smoothing to reduce noise and may therefore be more sensitive to small changes in overall activation level within regions.

With regard to the representational content of overlapping cortical regions and the basal ganglia, MVPA is thought to reflect sensitivity to distributed multidimensional information that is spatially distributed across multiple voxels (Davis et al., 2014). Thus, in overlapping cortical regions sequential rhythm and sequential order appear to be decodable when taking into account activation across multiple voxels. In the basal ganglia, however, each spiny neuron receives inputs from about 10,000 different afferents (Wilson, 1995). Consequently, a large amount of information that is widely distributed across the cortex is condensed into a markedly smaller number of neurons within the basal ganglia. It is not clear whether we still lack the resolution to identify more compact representation within the basal ganglia, or whether neural processes that appear dissociable at the cortex are not separately represented within neurons in the basal ganglia. The basal ganglia may be a region of integration for temporal and ordinal features, specifically in portions that are commonly active. Given its anatomical architecture of convergence and its functional role in action chunking, the basal ganglia may be well situated for integrating multiple movement features in order to concatenate multiple movements into a single action. Future research is needed to further address this issue.

## Conclusion

In summary, our results suggest that the representation of complex movement with differing rhythmic and ordinal structures is spatially distributed across cortical networks, including cortical regions that are commonly active for both complex movement tasks. Within the basal ganglia, small portions of the right putamen and caudate were more active for sequential rhythm, highlighting the role if the basal ganglia in rhythmic and temporal processes. There was also a large extent of bilateral putamen that was commonly activated by sequential rhythm and sequential order, which could not be reliably decoded based on the spatial activation patterns associated with each task. These findings support a multiplicative account (Kornysheva et al., 2013), in which rhythmic and ordinal feature are represented independently within the cortex, but integrated within the basal ganglia.

## Conflict of Interest Statement

The authors declare that the research was conducted in the absence of any commercial or financial relationships that could be construed as a potential conflict of interest.

## Footnotes

^1^http://www.fil.ion.ucl.ac.uk/spm

^2^http://fmri.wfubmc.edu/software/PickAtlas

^3^http://www.csie.ntu.edu.tw/~cjlin/libsvm

^4^http://www.sph.sc.edu/comd/rorden/mricron
